# Genetic variation in *Plethodon cinereus* and *Plethodon hubrichti* from in and around a contact zone

**DOI:** 10.1002/ece3.6653

**Published:** 2020-09-01

**Authors:** Robert B. Page, Claire Conarroe, Diana Quintanilla, Andriea Palomo, Joshua Solis, Ashley Aguilar, Kelly Bezold, Andrew M. Sackman, David M. Marsh

**Affiliations:** ^1^ Department of Life Sciences Texas A&M University‐San Antonio San Antonio TX USA; ^2^ Department of Biology Washington and Lee University Lexington VA USA

**Keywords:** approximate Bayesian computation, genetic differentiation, genetic diversity, mountaintop endemic, *Plethodon cinereus*, *Plethodon hubrichti*

## Abstract

Climate change poses several challenges to biological communities including changes in the frequency of encounters between closely related congeners as a result of range shifts. When climate change leads to increased hybridization, hybrid dysfunction or genetic swamping may increase extinction risk—particularly in range‐restricted species with low vagility. The Peaks of Otter Salamander, *Plethodon hubrichti*, is a fully terrestrial woodland salamander that is restricted to ~18 km of ridgeline in the mountains of southwestern Virginia, and its range is surrounded by the abundant and widespread Eastern Red‐backed Salamander, *Plethodon cinereus*. In order to determine whether these two species are hybridizing and how their range limits may be shifting, we assessed variation at eight microsatellite loci and a 1,008 bp region of *Cytochrome B* in both species at allopatric reference sites and within a contact zone. Our results show that hybridization between *P. hubrichti* and *P. cinereus* either does not occur or is very rare. However, we find that diversity and differentiation are substantially higher in the mountaintop endemic *P. hubrichti* than in the widespread *P. cinereus*, despite similar movement ability for the two species as assessed by a homing experiment. Furthermore, estimation of divergence times between reference and contact zone populations via approximate Bayesian computation is consistent with the idea that *P. cinereus* has expanded into the range of *P. hubrichti*. Given the apparent recent colonization of the contact zone by *P. cinereus*, future monitoring of *P. cinereus* range limits should be a priority for the management of *P. hubrichti* populations.

## Graphical abstract

This study uses population genetic approaches to assess whether hybridization between a severely range‐restricted mountaintop endemic salamander and a closely related widespread congener is contributing to the extinction risk of the former. While we find little to no evidence of hybridization, we do present evidence that the more common species is expanding into the range of the mountaintop endemic species. We discuss the conservation implications of this finding.

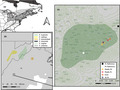

## 1 INTRODUCTION

As the Earth's climate changes, suitable habitat for many species will change in area and shift geographically (Milanovich, Peterman, Nibbelink, & Maerz, 2010; Parmesan & Yohe, 2003; Schloss, Nuñez, & Lawler, 2012). Consequently, range shifts resulting from species tracking suitable habitat are both expected and empirically well documented (Chen, Hill, Ohlemüller, Roy, & Thomas, 2011; Moritz et al., 2008; Parmesan et al., 1999; Tingley, Monahan, Beissinger, & Moritz, 2009). Climate‐driven range shifts can reshuffle communities in ways that amplify challenges to population persistence, and in some cases, increased interaction between closely related congeners may increase extinction risk via competition, hybrid dysfunction, or genetic swamping (Garroway et al., 2010; Gilman, Urban, Tewksbury, Gilchrist, & Holt, 2010; Walther, 2010). These kinds of scenarios are particularly likely for species with small distributions and low vagility because local extinction and global extinction are likely to be tightly intertwined and dispersing to find suitable habitat may present a significant challenge (Milanovich et al., 2010; Schloss et al., 2012). Similarly, species with highly specialized niches or narrow tolerances may face elevated extinction risk if their habitats become fragmented or unsuitable (Oliver et al., 2015; Urban, Tewksbury, & Sheldon, 2012).

Woodland salamanders in the genus *Plethodon* are a speciose (roughly 58 species; AmphibiaWeb, 2020) and morphologically conserved group of direct‐developing urodeles endemic to North America (Highton, 1995; Kozak & Wiens, 2010). The deepest split within *Plethodon* is geographic, and the two resulting groups are commonly referred to as the western and eastern clades (Highton, 1995). Most *Plethodon* species are in the eastern clade, and within this clade, three groups are usually recognized: (a) *P. glutinosus* group, (b) *P. wehrlei‐welleri* group, and (c) *P. cinereus* group (Wiens, Engstrom, & Chippindale, 2006). Within clades, *Plethodon* species may hybridize (Highton, 1995); in some cases, hybridization may be relatively rare or occur at only a few locations (Duncan & Highton, 1979), whereas in other cases hybridization is common in zones of sympatry (Chatfield, Kozak, Fitzpatrick, & Tucker, 2010; Hairston, Wiley, Smith, & Kneidel, 1992; Weisrock & Larson, 2006).

**FIGURE 1:**
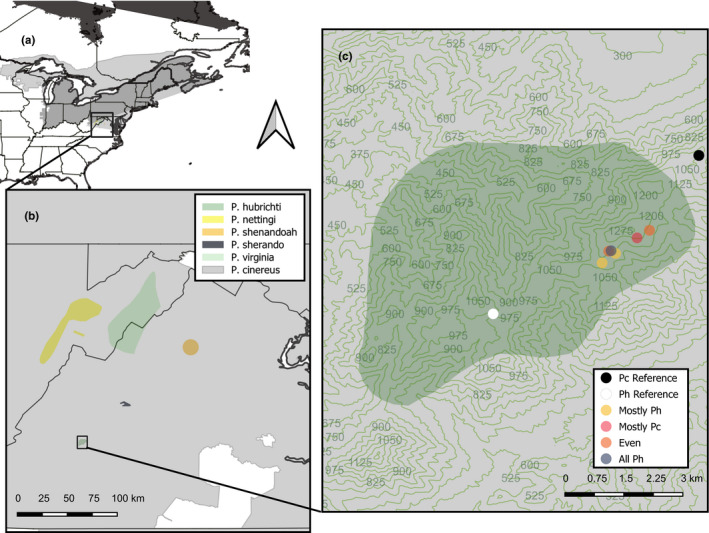
Map showing the range of *Plethodon* *cinereus* (a), the ranges of five mountaintop endemic salamanders from the *P. cinereus* species group (b), and the location of our sampling sites within and adjacent to the range of *P. hubrichti* (c). Contour lines in panel (c) are given in 75‐meter intervals. Distribution data are from the International Union for Conservation of Nature (IUCN)

Interestingly, clades of *Plethodon* typically include species that are very widespread and abundant, but also range‐restricted endemics with among the smallest ranges of any vertebrates in mainland North America (Highton, 1995). *Plethodon cinereus*, the Eastern Red‐backed Salamander, can reach densities of 2–3 individuals per m^2^ across large areas of Eastern North America from North Carolina to central Quebec (Hernández‐Pacheco, Sutherland, Thompson, & Grayson, 2019; Mathis, 1991; Figure 1a). In contrast, narrowly distributed endemics within this group are often species of conservation concern. For example, of the 10 species within the *P. cinereus* group, five (*P. shenandoah*, *P. sherando*, *P. hubrichti*, *P. nettingi*, and *P. virginia*) are range‐restricted mountaintop endemics that are listed as “near threatened” or “vulnerable” by the International Union for Conservation of Nature (IUCN, 2020; Figure 1b).

Population genetic data can potentially shed light on both the causes of range limits and how range shifts might affect species interactions, hybridization, and species persistence. For example, recent genetic findings suggest that *P. cinereus* may be tolerant of marginal habitat (Cameron, Page, Watling, Hickerson, & Anthony, 2019). As a result, *P. cinereus* may be well poised to undergo spatial shifts in range and abundance in response to climate change. The distributions of the five *P. cinereus* group mountaintop endemics are all nested within the range of *P. cinereus* (Figure 1). However, little is known about potential shifts in zones of parapatry between *P. cinereus* and closely related mountaintop endemics or increased hybridization resulting from any such shifts (but see Grant, Brand, De Wekker, Lee, & Wofford, 2018; Mulder, Cortes‐Rodriguez, Grant, Brand, & Fleischer, 2019). In the Southern Appalachian mountains where these species occur, climates are shifting to become warmer and drier, and in some cases, cloud heights are rising in elevation (Ingram, Dow, Carter, Anderson, & Sommer, 2013; Laseter, Ford, Vose, & Swift, 2012; Richardson, Denny, Siccama, & Lee, 2003) Each of these aspects of climate change, alone or in combination, has the potential to substantially shift salamander distributions and the nature of their interspecific interactions (Grant et al., 2018; Milanovich et al., 2010; Walls, 2009).

We present a population genetics study from in and around a contact zone between the Peaks of Otter Salamander (*P. hubrichti*) and the Eastern Red‐backed Salamander (*P. cinereus)* that was designed to determine: (a) whether *P. hubrichti* and *P. cinereus* are hybridizing and (b) whether contact zone populations from both species are expanding, contracting, or stable. We supplement these analyses with an experimental homing study to compare the relative mobility of the two species in order to better interpret the population genetics results. Finally, we discuss the evolutionary and conservation implications of our findings in terms of the extraordinarily small range of *P. hubrichti* (Figure 1), low vagility of woodland salamanders, and challenges faced by mountaintop endemics in an era of climate change.

## 2 MATERIALS AND METHODS

### 2.1 Tissue collection & field sites

*Plethodon hubrichti* and *P. cinereus* are lungless, forest‐dwelling amphibians that require access to moist microhabitat to facilitate cutaneous respiration (Heatwole, 1962; Reichenbach & Brophy, 2017). As such, during spring, summer, and fall, both species frequently occupy cover objects, such as rocks and logs, on the forest floor and forage in leaf litter following rainfall. We collected tissue for genetic analysis by searching under cover objects and capturing animals by hand. After salamanders were caught, we induced tail autotomy by lightly clasping the tail with forceps approximately 1 cm from the tip. Salamanders were then immediately released at their sites of capture and tail tips were placed in 90%–100% molecular biology grade ethanol until returning to the lab, whereupon they were stored at −80°C.

Tissue samples were collected from three areas within the Apple Orchard Mountain region of the George Washington National Forest (Figure 1c) between May 10, 2017, and August 07, 2017. The most westerly locale (“Floyd's Field”) was sampled as a reference site for *P. hubrichti,* as this site is approximately 3 km from any known contact zone with *P. cinereus*. From this site, we obtained 24 samples of *P. hubrichti*. Similarly, the most easterly locale (“Thunder Ridge”) was sampled as a reference site for *P. cinereus* and is situated at a similar distance from the Apple Orchard Mountain contact zone. From this site, we took 24 samples of *P. cinereus*. The sites at intermediate longitudes (Figure 1c) occur within an approximately 2‐km‐wide contact zone where both species are found. In general, we sampled salamanders at random with respect to morphology within the contact zone sites and identified them to species based on morphological characteristics. *Plethodon hubrichti* (*N* = 48) were distinguished by a solid black venter and a bronze‐colored stripe, whereas *P. cinereus* (*N* = 60) were distinguished by a “salt and pepper” venter and an orange stripe. In addition, 20 salamanders (7 *P. hubrichti* and 13 *P. cinereus*) were selected for inclusion from a larger contact zone sample taken during the same time period. These 20 salamanders were selected specifically based on their unusual morphology—for example, salamanders that appeared to be unstriped *P. hubrichti* or salamanders that appeared to be *P. cinereus* but had stripes that were yellowish rather than orange. Randomly sampled salamanders were used to make inferences about contact zone populations (e.g., with respect to frequency of hybridization), whereas salamanders selected as morphological outliers provide a stronger test of whether hybridization occurs at all.

### 2.2 DNA isolation, mtDNA sequencing, and mtDNA sequence analysis

DNA was isolated from tail tissue using the Qiagen DNeasy Blood and Tissue Kit according to the manufacturer's instructions. We then used the primers described in Bayer, Sackman, Bezold, Cabe, and Marsh (2012; PcCytB‐F‐T3 5′‐AATTAACCCTCACTAAAGGGCTCAACCAAAACCTTTGAC C‐3′ and PcCytB‐R 5′‐TAGCCCCCAATTTTGGTTT ACA‐3′) to amplify a portion of the mitochondrial locus, *Cytochrome B* (*CYTB*). Sequencher version 5.4 (Gene Codes Corporation) was used to generate a 1,008 bp alignment from 25 *P. hubrichti* sequences (7 from the reference site, and 18 from the contact zone) and 23 *P. cinereus* sequences (3 from the reference site and 20 from the contact zone). We then used MEGA version 10.0.5 (Kumar, Stecher, Li, Knyaz, & Tamura, 2018) to calculate descriptive statistics for genetic diversity and the R package *pegas* (Paradis, 2010) to construct haplotype networks for these sequences.

### 2.3 Microsatellite genotyping

The microsatellite loci developed from *P. cinereus* by Cameron, Anderson, and Page (2017) were screened for cross‐amplification in *P. hubrichti*, and eight loci (Pc4, Pc7, Pc15, Pc17, Pc20, Pc22, Pc28, and Pc37) that reliably amplified were identified. Genotyping reactions followed the methodology outlined in Cameron et al. (2017). Briefly, PCRs were 25 µl in volume and contained 1× buffer, 10–20 ng of template DNA, 1.5 mM MgCl_2_, 0.2 mM of each dNTP, 0.8 µM of non‐M13(−21)‐tagged primer, 0.8 µM of 6‐FAM‐ or HEX‐labeled M13(−21) primer, 0.2 µM of M13(−21)‐tagged primer, and 0.625 units of GoTaq polymerase (Promega). Thermal cycler conditions were 92°C for 2 min, followed by 25 cycles of (a) 94°C for 30 s, (b) 62°C for 30 s, decreasing by 0.3°C per cycle, and (c) 72°C for 40 s. To facilitate our nested PCR approach (see Schuelke, 2000), we performed eight additional cycles of (a) 94°C for 30 s, (b) 53°C for 30 s, and (c) 72°C for 40 s, followed by a final extension step of 72°C for 30 min. When performing these reactions, several DNA samples were aliquoted into more than one well within our template DNA microtiter plates, which enabled us to generate replicate PCRs that were used to estimate genotyping error rates.

All PCRs were screened for successful amplification via 2% agarose gel electrophoresis and successful reactions were shipped to the Arizona State University DNA Lab where they were subjected to capillary electrophoresis using an ABI 3730 and GENESCAN LIZ 600 as an internal sizing standard. Standard curve fitting, genotype scoring, and binning were performed using the microsatellite plugin for GENEIOUS, version R9 (Biomatters). Of the original 176 samples, 170 amplified successfully and yielded microsatellite genotypes, 18 of which were from individuals sampled specifically for having unusual morphology.

### 2.4 Analysis of microsatellite data

#### 2.4.1 Quality control and summary statistics

Summary statistics including number of alleles, effective number of alleles, observed heterozygosity (H_O_), and expected heterozygosity (H_E_) were computed in GelAlEx (Peakall & Smouse, 2012) for both species' reference and contact zone populations (i.e., analytical units resulting from pooling the nonreference site locales for each respective species) and for each species irrespective of presumptive subdivision. We also used POPGENREPORT (Adamack & Gruber, 2014) to calculate allelic richness (*A*_R_) values for the reference and contact zone populations of both species and for each species irrespective of subdivision. We then used GENEPOP for R (Rousset, 2008) to assess whether the reference and contact zone populations of the two respective species exhibited significant departures from Hardy–Weinberg proportions and genotypic equilibrium. GENEPOP was also used to calculate the Weir and Cockerham (1984) estimator of *F*_IS_ for the reference and contact zone populations of both respective species. Finally, we assessed the evidence for null alleles, large allele dropout, and scoring errors in the reference and contact zone populations of both respective species using MICROCHECKER, Version 2.2.3 (Van Oosterhout, Hutchinson, Wills, & Shipley, 2004).

#### 2.4.2 Differentiation, admixture, and population structure

We used our microsatellite data to conduct several analyses that examine patterns of genetic variation between and within *P. cinereus* and *P. hubrichti*. First, we used STRUCTURE 2.3.4 (Falush, Stephens, & Pritchard, 2003; Pritchard, Stephens, & Donnelly, 2000) to infer the optimal partition of multilocus genotypes from both species under the assumptions of Hardy–Weinberg and linkage equilibria and to assess admixture between species. We used the admixture model with correlated allele frequencies to allow for hybridization between species and the possibility that clusters within species trace to a common ancestral population. Because plethodontids can exhibit detectable substructure over small spatial scales (e.g., Cabe et al., 2007), we examined a range of *K* values that allow for the possibility of subdivision within one or both species (*K* = 1–5) and inspected mean Ln P(D) ± *SD* and deltaK (Evanno, Regnaut, & Goudet, 2005) plots based on replicate runs (*n* = 15 for each value of *K*) to determine the optimal value of *K*. When running STRUCTURE, we used a burn‐in period 250,000 MCMC steps followed by an additional 250,000 sampled MCMC steps. We also performed this analysis separately on the *P. cinereus* and *P. hubrichti* genotypes, respectively, with the exception that we assessed a smaller range of *K* values (i.e., *K* = 1–4) when conducting these species‐specific analyses. For all three of these analyses, we used STRUCTURE HARVESTER (Earl & vonHold, 2012) to visualize and summarize the results of our replicate STRUCTURE runs and CLUMPP (Jakobsson & Rosenberg, 2007) to align cluster assignments across runs.

To complement our analyses in STRUCTURE, we used the Bayesian method implemented in NewHybrids Version 1.1 to estimate the parameters of the model described by Anderson and Thompson (2002). This approach enabled us to probabilistically assign individuals to six classes expected to be present following two generations of interbreeding: pure *P. cinereus*, pure *P. hubrichti*, F_1_ hybrid, F_2_ hybrid, backcrossed hybrid from a mating between an F_1_ and *P. cinereus*, and backcrossed hybrid from a mating between an F_1_ and *P. hubrichti*. When running NewHybrids, we initially assessed convergence using the real‐time graphical displays available in the program, which suggested a burn‐in period of 100,000 sweeps, followed by 200,000 recorded sweeps would be sufficient to ensure convergence. We then used this burn‐in and number of sampled steps to conduct five replicate runs with uniform priors and five replicate runs with Jeffreys priors, treating all individuals as part of the “mixture” (Anderson, 2003; Anderson & Thompson, 2002) regardless of sampling locale. Lastly, we used the “s” and “z” options available in the program to designate individuals sampled at Thunder Ridge as pure *P. cinereus* and individuals sampled at Floyd's Field as pure *P. hubrichti*, which enabled us to use animals from reference sites to estimate allele frequencies for the respective species, while excluding them from the mixture (Anderson, 2003). When implementing this approach, we used the same number of burn‐in and sampled sweeps as before and conducted five replicate runs based on uniform priors and five replicate runs based on Jeffreys priors.

Because empirical data may not exhibit Hardy–Weinberg or linkage equilibria, the two main assumptions of Bayesian clustering algorithms like STRUCTURE and NewHybrids, it is important to assess differences between and within species using alternative approaches. To this end, we used discriminant analysis of principal components (DAPC; Jombart, Devillard, & Balloux, 2010) to partition multilocus genotypes from both species. We used the *adegenet* package for R (Jombart, 2008) to infer *K* by computing *K*‐means clustering solutions for *K* = 1–10 and assessing these solutions via the Bayesian information criterion (BIC). DAPC was then performed using the assignments from the *K*‐means routine as prior group assignments. Before performing DAPC, the optimal number of principal components to retain was assessed via the cross‐validation procedure described by Jombart and Collins (2015).

We also used GenAlEx to perform an analysis of molecular variance (AMOVA; Excoffier, Smouse, & Quattro, 1992) that partitioned genetic variation among species, among reference and contact zone populations (i.e., analytical units generated by pooling across locales within the zone of sympatry) within species, among individuals within populations, and within individuals. To complement this analysis, we also used the QDiver module within GenAlEx to implement an analogous “different is different” hierarchical partition of diversity as recently described by Smouse, Banks, and Peakall (2017). We also used GenAlEx to calculate locus‐by‐locus and overall estimates of Joust's *D* (*D*_EST_; Jost, 2008; Meirmans & Hedrick, 2011) between *P. cinereus* and *P. hubrichti* irrespective of sampling locale. Finally, we used GenAlEx to calculate locus by locus, global, and pairwise *G*′_ST_ and D_EST_ (Meirmans & Hedrick, 2011) estimates between the reference and contact zone populations (as defined above) of both respective species. All significance tests performed in GenAlEx were based on 9,999 permutations.

#### 2.4.3 Gene flow

We used our microsatellite data to assess gene flow between the reference and contact zone populations (i.e., analytical units generated by pooling across locales within the zone of sympatry) of both respective species using MIGRATE version 3.7.2 (Beerli, 2009). MIGRATE uses a coalescent framework and Bayesian estimation scheme to produce estimates of *θ* (4*N*_e_ *µ*, where *µ* is the mutation rate) for each population and asymmetrical mutation scaled migration rates (*M* = *m*/*µ*, where m is the proportion of immigrants) for population pairs. Thus, MIGRATE can also be used to obtain asymmetrical estimates of 4*N*_e_ *m* by taking the product of *θ* and *M* estimates, which is the approach that we have taken. We used the Brownian motion model and estimated the relative mutation rate of each locus from our data. Metropolis‐Hastings sampling was used for four replicate long chains for 25,000,000 iterations with a burn‐in of 5,000,000, and a sampling interval of 500. We assumed uniform priors for *θ* (lower bound = 0, upper bound = 200) and *M* (lower bound = 0, upper bound = 3,000) and used *F*_ST_ to determine the initial estimates of both parameters. To assess convergence, we compared output from multiple runs, examined posterior distributions, and effective sample sizes (ESS) for all parameter estimates and considered estimates with ESS > 1,000 acceptable.

#### 2.4.4 Bottleneck testing

We used our microsatellite data and the program BOTTLENECK (Piry, Luikart, & Cornuet, 1999) to assess whether genetic signatures associated with recent population reductions are present in the reference and contact zone populations (i.e., analytical units generated by pooling across locales within the zone of sympatry) of *P. hubrichti* and *P. cinereus*. BOTTLENECK is based on the observation that following population reductions, H_E_ becomes larger than the heterozygosity expected at mutation–drift equilibrium (H_EQ_). As such, the program conducts simulations and tests to assess whether the difference between H_E_ and H_EQ_ is statistically significant. We tested for departures under a two‐phase mutation model (TPM) with 70% stepwise mutation model (SMM) and a variance of 30, which is recommended by Di Rienzo et al. (1994) for microsatellites. We ran 1,000,000 iterations and used the Wilcoxon signed rank and mode shift tests to assess departures between H_E_ from H_EQ_ for statistical significance.

#### 2.4.5 Demographic modeling

**FIGURE 2:**
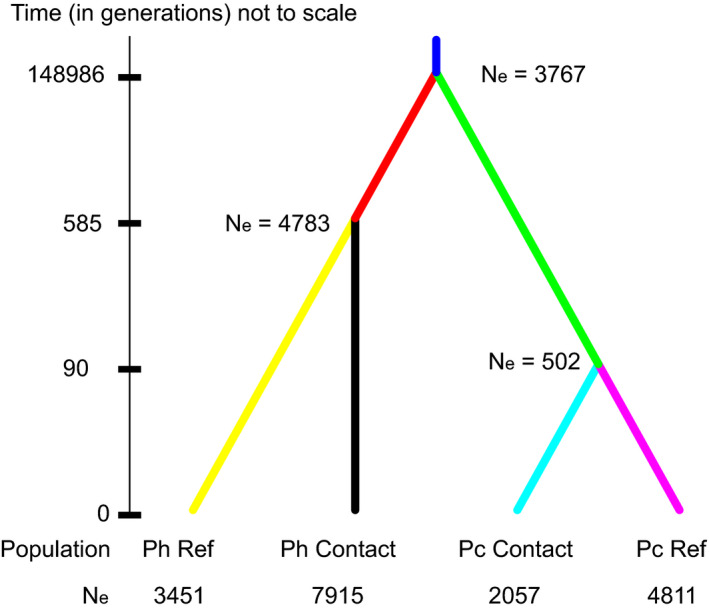
Illustration of the demographic history estimated with DIYABC. Effective population sizes and divergence times (in generations, not to scale) were derived from the median posterior parameter distributions. Population sizes given at the nodes represent the inferred ancestral population sizes before splits. Ph Ref, Floyd's Field; Ph Contact, *P. hubrichti* contact zone; Pc Contact, *P. cinereus* contact zone; and Pc Ref, Thunder Ridge

We used approximate Bayesian computation (ABC) as implemented in the program DIYABC (Cornuet et al., 2014) to fit a demographic history for the *P. hubrichti* and *P. cinereus* populations using all eight microsatellite loci from 170 individuals as well as *CYTB* sequences from 45 individuals (three *P. hubrichti* from the contact zone that were sequenced at *CYTB* had no corresponding microsatellite data and were therefore dropped from this analysis; Figure 2). Effective population sizes were estimated for the current *P. hubrichti* and *P. cinereus* reference and contact zone (i.e., analytical units generated by pooling across locales within the zone of sympatry) populations. In addition, we estimated population sizes and divergence times (in generations) for ancestral *P. hubrichti* and *P. cinereus* populations, as well as the common ancestral population of all four present‐day populations. All eight microsatellite loci were analyzed as a single group, using a generalized stepwise model with the default priors for mutation rate, including a mean mutation rate of 5 × 10^–4^ (Garza & Williamson, 2001), consistent with the rate used in our MIGRATE analyses (see MIGRATE results below). The mitochondrial data were analyzed as a single group, using the Kimura 2 Parameters mutation model, with uniform priors for mean mutation rate and individual locus mutation rates ranging from 1 × 10^–9^ to 1 × 10^–6^. Uniform priors were used for the *N*_e_ and divergence time parameters of the model. We performed 10^6^ simulations, and posterior parameter distributions were generated from the 10^4^ simulated data sets most closely matching the observed data, as calculated from the mean number of alleles, mean genic diversity, mean Garza‐Williamson's *M* (Garza & Williamson, 2001), pairwise *F*_ST_ (Weir & Cockerham, 1984), and genetic distance between populations (δμ)^2^ (Goldstein, Linares, Cavalli‐Sforza, & Feldman, 1995). A total of 500 pseudo‐observed datasets (pods) were simulated with values drawn from the posterior distributions to assess the precision and bias of the posterior parameters (Table 1).

**TABLE 1:** Bias estimates for the root of the relative mean integrated square error (RRMISE), relative median absolute deviation (RMedAd), average relative bias, factor 2 score of the modes of the posterior distributions, and highest density probability intervals (HDPI) of the demographic history estimated in DIYABC

Parameter	Median	Mode	90% HPDI	RRMISE	RMedAd	Avg. Rel. Bias	Factor 2
*N*_e_ Ph Ref	3.90e3	2.19e3	412–7,429	1.907	0.891	0.262	0.806
*N*_e_ Ph Contact	7.92e3	8.72e3	5,368–9,999	0.540	0.343	−0.151	0.970
*N*_e_ Pc Ref	4.81e3	3.94e3	1,302–9,009	1.449	0.831	0.116	0.778
*N*_e_ Pc Contact	2.06e3	9.59e2	186–5,675	5.299	2.069	1.020	0.480
*N*_e_ Ph Anc.	4.78e3	4.15e3	1,290–8,709	1.287	0.776	0.317	0.784
*N*_e_ Pc Anc.	5.03e2	2.42e2	14–1,414	2.504	1.262	0.469	0.650
*N*_e_ Anc.	3.77e3	2.26e2	19–8,477	20.610	4.596	0.320	0.602
*T*_Div_ Ph	5.85e2	7.23e2	212–992	1.465	0.688	−0.170	0.808
*T*_Div_ Pc	8.96e1	3.21e1	1–369	7.074	2.195	0.738	0.558
*T*_Div_ Anc.	1.49e5	7.33e4	1.09e4–4.54e5	2.2129	1.150	0.450	0.674

Note

Parameters are given for *N*_e_ of the reference and contact zone populations of *P. hubrichti* (Ph) and *P. cinereus* (Pc), the ancestral populations for each species, and the ancestral population of all four populations, as well as for the estimated divergence times for each branch in the demographic history.

### 2.5 Homing experiment

Patterns of genetic differentiation among sites depend on the mobility of a species. Although one small‐scale tracking study suggested that *P. hubrichti* and *P. cinereus* generally have similar movement behavior (Goff, 2015), less is known about their dispersal ability beyond the scale of a few meters. Bernardo, Ossola, Spotila, and Crandall (2007) hypothesized that mountaintop endemic salamanders should evolve low metabolic rates as an adaptation to high‐elevation environments. These lower metabolic rates would then lead to reduced dispersal ability and higher levels of genetic differentiation among subpopulations (Bernardo et al., 2007). Based on this scenario, one might expect *P. hubrichti* to be less mobile than *P. cinereus*, and to have higher levels of genetic differentiation as a consequence.

Unfortunately, it is difficult to determine movement rates directly in woodland salamanders because they are too small for radio transmitters, and recapture rates for marked animals are typically very low even with very large samples (Bailey, Simons, & Pollock, 2004; Gillette, 2003). However, displaced salamanders are effective at homing back to their capture location (Kleeberger & Werner, 1982), so experimentally releasing salamanders and estimating return rates provides an indirect method to assess relative movement ability across different conditions. Although homing is obviously not equivalent to dispersal under natural conditions, return rates in homing experiments do decline as a function of distance and landscape barriers (Marsh, Milam, Gorham, & Beckman, 2005; Marsh, Thakur, Bulka, & Clarke, 2004), suggesting that these rates have some biological relevance.

In order to test the relative movement ability of *P. cinereus* and *P. hubrichti*, we experimentally displaced salamanders of both species from the same plot and observed frequency of homing as a function of species and distance. This plot contained a grid of 156 cover objects (rocks and logs) separated by 4 m and numbered with metal tags (see Marsh et al., 2019 for details). We periodically surveyed this plot between June 2018 and October 2019 and captured any juvenile or adult *P. cinereus* or *P. hubrichti* that we encountered (hatchlings were excluded). We measured the snout‐vent length of these salamanders, but did not record their sex, although it is possible that dispersal rates would differ between males and females (Muñoz, Miller, Sutherland, & Grant, 2016). Salamanders were individually marked with fluorescent elastomer tags (Northwest Marine Technology; Davis & Ovaska, 2001) and randomly assigned to one of three treatments: (a) controls, which were marked and then returned to the cover object under which they were captured, (b) salamanders displaced 15 m in a random direction, and (c) salamanders displaced 30 m in a random direction. For the latter two treatments, salamanders were released underneath ceramic floor tiles to provide them with temporary cover. Over the course of the study, we resurveyed the plot and recorded any salamanders that had returned to their original location. We then used logistic regression to compare the probability of return between species and between distances (i.e., 15 m vs. 30 m). These analyses were carried out using the glm function in R version 3.6.

## 3 RESULTS

### 3.1 mtDNA sequence analysis

**FIGURE 3:**
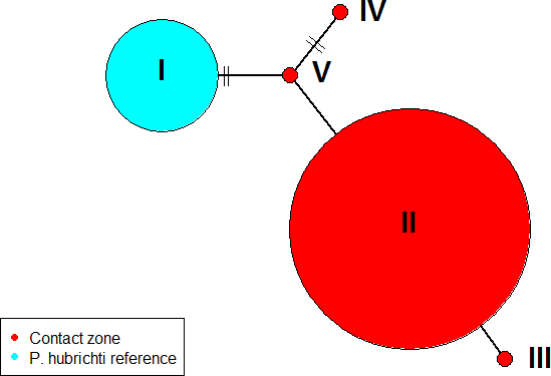
Network for the five *cytochrome B* haplotypes recovered from *Plethodon* *hubrichti*

The 23 *CYTB* sequences for *P. cinereus* were identical (i.e., no variable sites). In contrast, for the 25 *P. hubrichti* sequences, there were 9 variable sites and 5 distinct haplotypes. One haplotype (I) corresponded to the 7 samples from the *P. hubrichti* reference site, whereas the others (II–V) were found across the contact zone (Figure 3) with 15 individuals of haplotype II and one each from haplotypes III, IV, and V. The coefficient of differentiation among sites was 0.596, the diversity within subpopulations was 0.0010, and the diversity for the entire population was 0.0026.

### 3.2 Microsatellite analyses

#### 3.2.1 Quality control and summary statistics

Examination of technically replicated genotyping reactions indicated that genotyping error rates are low and ranged from zero disagreements among 22 paired replicates at Pc22 to one disagreement among 11 paired replicates at Pc28. Across all loci, there were 4 disagreements among 123 paired replicates, leading to an overall genotyping error rate estimate of 3.25%.

Summary statistics for the respective reference and contact zone populations of *P. hubrichti* and *P. cinereus* and both species ignoring presumptive subdivision are presented in Table 2. Upon adjusting for multiple testing (Holm, 1979) by treating the tests associated with each population as a family of tests, there was no evidence of genotypic disequilibrium between any pair of loci within the reference and contact zone populations of either species. However, after correcting for multiple testing in analogous fashion, there was evidence of departure from Hardy–Weinberg proportions at Pc7 and Pc28 in the *P. hubrichti* contact zone population. Similarly, Pc7 and Pc37 exhibited statistical departures from Hardy–Weinberg proportions in the *P. cinereus* contact zone population, and Pc28 departed from Hardy–Weinberg proportions in Thunder Ridge. MICROCHECKER flagged Pc28 and Pc37 in the *P. hubrichti* contact zone population, Pc28 in Thunder Ridge, and Pc37 in the *P. cinereus* contact zone population as potentially harboring null alleles. However, prior microsatellite surveys of *P. cinereus* from contiguous habitat elsewhere in Virginia have shown that 82% of individuals collected from 50‐m^2^ plots separated by 200 m can be correctly assigned and that this proportion rises to 94% when plots of this size are separated by 2 km (Cabe et al., 2007). Thus, modest Wahlund effects would not be a surprising consequence of pooling the contact zone locales (Figure 1c) into a single analytical unit for each species. Moreover, with one exception (see NewHybrid results below), two sets of exploratory analyses, one of which was based on a dataset composed of Pc4, Pc15, Pc20, and Pc22, and the other of which was based on a dataset with Pc37 removed, gave qualitatively similar results to analyses based on all eight loci. Therefore, the analyses presented herein are based on all eight loci, unless otherwise indicated.

**TABLE 2:** Summary statistics for microsatellite loci

Locus	*N*	*A*	*A*_E_	*A*_R_	*H*_E_	*H*_O_	*F*_IS_
*Plethodon hubrichti* reference site (Floyd's Field)							
Pc4	22	9	6.245	8.346	0.840	0.864	−0.005
Pc7	21	5	2.782	4.352	0.641	0.667	−0.016
Pc15	21	9	5.654	8.147	0.823	0.810	0.041
Pc17	24	6	4.535	5.844	0.780	0.792	0.006
Pc20	21	2	1.153	1.970	0.133	0.048	0.655
Pc22	21	5	3.379	4.668	0.704	0.619	0.145
Pc28	18	8	3.100	7.467	0.677	0.667	0.045
Pc37	21	1	1.000	1.000	0.000	0.000	NA
Mean	21.125	5.625	3.481	5.224	0.575	0.558	0.124
*SEM*	0.581	1.068	0.677	0.975	0.114	0.120	0.091
*Plethodon hubrichti* contact zone							
Pc4	47	14	8.033	10.634	0.876	0.915	−0.034
Pc7	47	5	2.737	4.710	0.635	0.638	0.005
Pc15	49	13	7.072	9.444	0.859	0.939	−0.083
Pc17	48	11	5.364	8.582	0.814	0.750	0.089
Pc20	48	2	1.021	1.341	0.021	0.021	NA
Pc22	47	11	5.323	8.641	0.812	0.745	0.094
Pc28	44	13	3.748	9.432	0.733	0.545	0.267
Pc37	47	2	1.043	1.576	0.042	0.000	1.000
Mean	47.125	8.875	4.293	6.795	0.599	0.569	0.191
*SEM*	0.515	1.787	0.925	1.314	0.127	0.130	0.142
*Plethodon cinereus* reference site (Thunder Ridge)							
Pc4	22	10	5.204	8.535	0.808	0.773	0.067
Pc7	21	3	2.930	3.000	0.659	0.714	−0.060
Pc15	22	3	2.082	2.654	0.520	0.591	−0.114
Pc17	22	2	1.146	1.962	0.127	0.136	−0.050
Pc20	21	3	1.213	2.646	0.176	0.190	−0.060
Pc22	20	2	1.051	1.701	0.049	0.050	NA
Pc28	19	5	1.473	4.175	0.321	0.158	0.528
Pc37	20	3	1.107	2.401	0.096	0.050	0.500
Mean	20.875	3.875	2.026	3.384	0.344	0.333	0.116
*SEM*	0.398	0.934	0.508	0.782	0.101	0.108	0.105
*Plethodon cinereus* contact zone							
Pc4	68	10	6.835	7.973	0.854	0.809	0.060
Pc7	73	6	2.551	4.388	0.608	0.534	0.128
Pc15	69	4	2.551	3.240	0.608	0.696	−0.137
Pc17	72	4	1.058	1.885	0.054	0.042	0.242
Pc20	70	3	1.371	2.562	0.271	0.300	−0.101
Pc22	69	3	1.315	2.240	0.239	0.275	−0.143
Pc28	67	2	1.030	1.440	0.029	0.030	−0.008
Pc37	69	4	1.195	2.928	0.163	0.000	1.000
Mean	69.625	4.500	2.238	3.332	0.353	0.336	0.130
*SEM*	0.706	0.886	0.693	0.735	0.106	0.111	0.133
*Plethodon hubrichti* ignoring subdivision							
Pc4	69	14	7.955	13.269	0.874	0.899	−0.021
Pc7	68	5	3.085	4.998	0.676	0.647	0.050
Pc15	70	15	8.235	14.010	0.879	0.900	−0.017
Pc17	72	13	6.194	12.493	0.839	0.764	0.096
Pc20	69	3	1.060	2.671	0.057	0.029	0.494
Pc22	68	11	5.955	10.238	0.832	0.706	0.159
Pc28	62	16	3.714	14.851	0.731	0.581	0.213
Pc37	68	2	1.030	1.914	0.029	0.000	1.000
Mean	68.250	9.875	4.653	9.306	0.614	0.566	0.247
*SEM*	1.01	2.004	1.011	1.875	0.127	0.127	0.122
*Plethodon cinereus* ignoring subdivision							
Pc4	90	13	7.334	11.200	0.864	0.800	0.079
Pc7	94	6	2.698	5.423	0.629	0.574	0.093
Pc15	91	4	2.451	3.566	0.592	0.670	−0.127
Pc17	94	5	1.078	3.814	0.073	0.064	0.127
Pc20	91	4	1.334	3.485	0.250	0.275	−0.092
Pc22	89	3	1.253	2.821	0.202	0.225	−0.108
Pc28	86	5	1.112	4.090	0.101	0.058	0.429
Pc37	89	6	1.175	4.937	0.149	0.011	0.926
Mean	90.500	5.750	2.304	4.917	0.358	0.335	0.166
*SEM*	0.945	1.098	0.753	0.944	0.104	0.108	0.126

Note

*A*_R_ estimates for the two species irrespective of subdivision were standardized to a sample size of 62, while *A*_R_ estimates for the reference and contact zone populations were standardized to a sample size of 18. NA = quantity that could not be estimated.

Abbreviations: *A*, number of alleles; *A_E_*, effective number of alleles; *A*_R_, allelic richness; *F*_IS_, Weir and Cockerham's inbreeding coefficient estimator; H_E_, Hardy–Weinberg expected heterozygosity; *H*_O,_ observed heterozygosity; *N*, number of individuals sampled.

#### 3.2.2 Differentiation, admixture, and population structure

**FIGURE 4:**
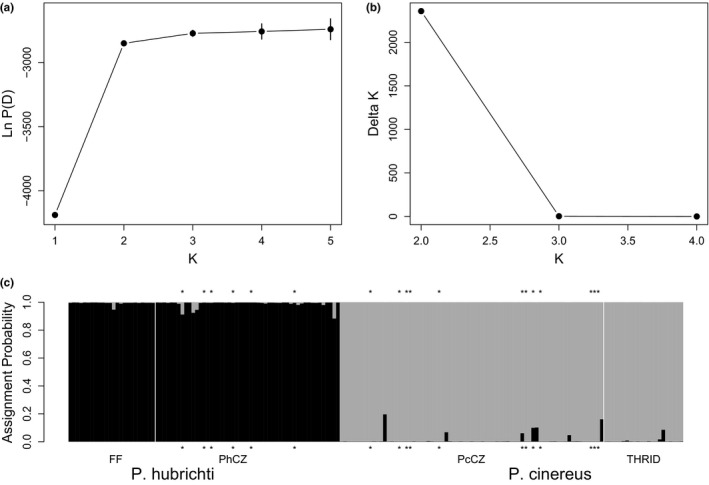
Results of the global analysis performed in STRUCTURE, including mean Ln P(D) ± *SD* across 15 replicate runs for each value of *K* (a), Δ*K* as a function *K* (b), and the Q‐matrix as a stacked bar chart (c). In Panel c, asterisk denotes individuals with unusual morphology, white vertical lines denote breaks between locales within species, FF, Floyd's Field; PhCZ, *P. hubrichti* contact zone; PcCZ, *P. cinereus* contact zone; and THRID = Thunder Ridge

Examination of mean Ln P(D) ± *SD* and deltaK plots resulting from the global analysis that we performed in STRUCTURE identified *K* = 2 as the optimal partition of *P. hubrichti* and *P. cinereus* genotypes (Figure 4a,b). As can be seen in Figure 4c, all *P. hubrichti* genotypes were assigned to one cluster and all *P. cinereus* genotypes were assigned to the other. Moreover, the minimum estimated proportion of ancestry for *P. hubrichti* samples in the *P. hubrichti* cluster was 0.883 and the minimum estimated proportion of ancestry for *P. cinereus* samples in the *P. cinereus* cluster was 0.804. Most likely, nonunity admixture coefficients are largely attributable to size homoplasy, which is commonly observed among species (Estoup, Tailliez, Cornuet, & Solignac, 1995; Primmer & Ellegren, 1998) and positively associated with divergence time (Bhargava & Fuentes, 2010; Estoup et al., 1995).

**FIGURE 5:**
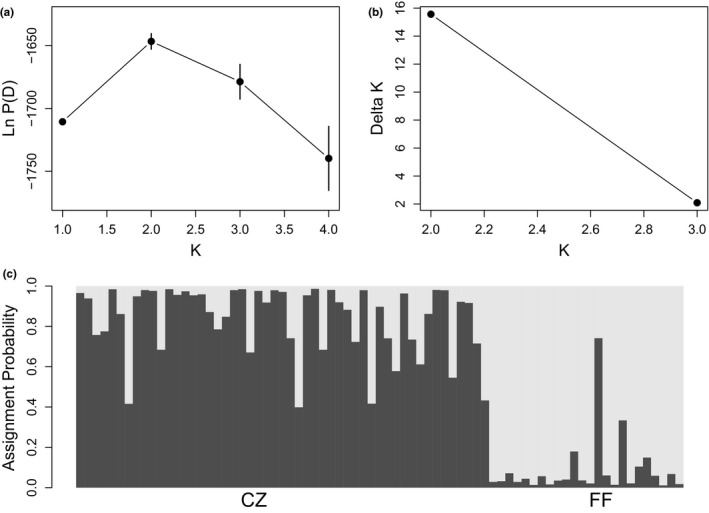
Results of the STRUCTURE analysis performed on the *Plethodon* *hubrichti* genotypes, including Ln P(D) ± *SD* across 15 replicate runs for each value of *K* (a), Δ*K* as a function *K* (b), and the Q‐matrix as a stacked bar chart (c). In panel c, CZ = contact zone, and FF = Floyd's Field

**FIGURE 6:**
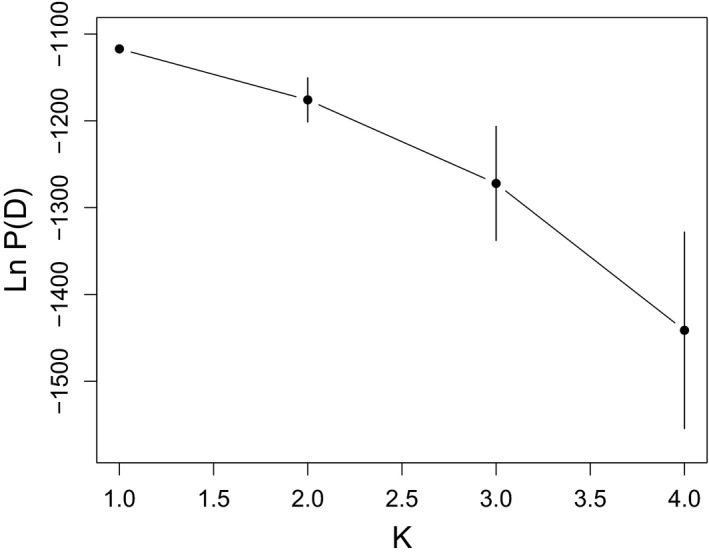
Ln P(D) ± *SD* across 15 replicate STRUCTURE runs on the *Plethodon* *cinereus* genotypes

Our STRUCTURE analysis of the *P. hubrichti* genotypes suggested subdivision within this species as *K* = 2 was identified as the optimal partition (Figure 5a,b). As can be seen in Figure 5c, assignment patterns among the *P. hubrihcti* genotypes suggest differentiation between the reference locale (Floyd's Field) and sites within the contact zone. Conversely, an analogous analysis of *P. cinereus* genotypes failed to detect substructure, as *K* = 1 was identified as the optimal partition of these data (Figure 6).

**FIGURE 7:**
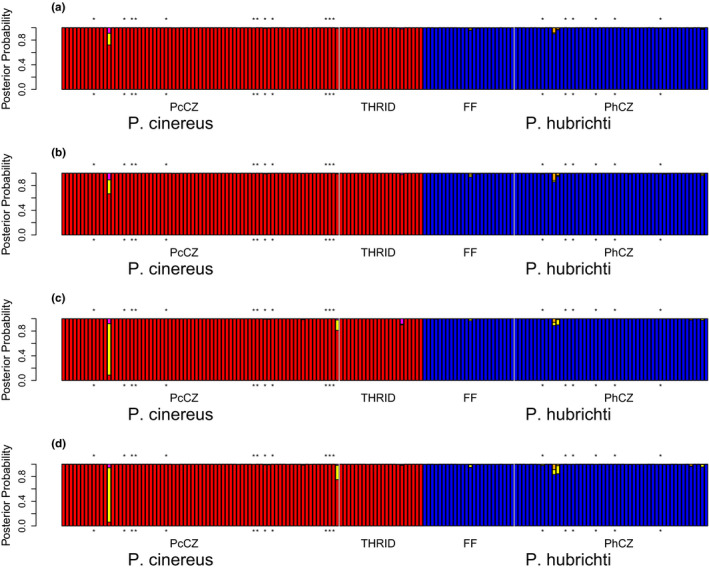
Posterior probabilities of the various genetic categories examined with NewHybrids using uniform priors and including individuals from reference sites in the mixture (a), using uniform priors and excluding individuals from reference sites from the mixture (b), using Jeffreys priors and including individuals from reference sites in the mixture (c), and using Jeffreys priors and excluding individuals from reference sites from the mixture (d). Note that the program estimates posterior probabilities for individuals from reference sites even when they are excluded from the mixture. Blue = pure *P. hubrichti*, red = pure *P. cinereus*, green = F_1_ hybrid, yellow = F_2_ hybrid, orange = backcrossed hybrid from a mating between an F_1_ and *P. hubrichti*, magenta = backcrossed hybrid from a mating between an F_1_ and *P. cinereus*. *Individuals with unusual morphology. Vertical white lines delineate locales within species. PcCZ, *P. cinereus* contact zone; THRID, Thunder Ridge; FF, Floyd's Field, and PhCZ, *P. hubrichti* contact zone

The analyses we performed in NewHybrids largely agreed with the results of our global analysis in STRUCTURE (Figure 7). Irrespective of whether individuals from reference sites were excluded from the mixture, there was little evidence for the presence of hybrids in our sample under uniform priors (Figure 7a,b). However, under Jeffreys priors, one individual from the contact zone identified as a *P. cinereus* without unusual morphology was flagged as a F_2_ hybrid (Figure 7c,d). The parameter estimates generated by NewHybrids are known to be sensitive to the priors (Anderson & Thompson, 2002), and these discrepancies do not appear to be due to convergence issues as inspection of real‐time graphics and congruence between independent runs both indicate our runtimes were sufficient. However, the individual flagged as a hybrid in these runs was scored as homozygous for an allele at Pc37 that was otherwise only found in *P. hubrichti*. Rerunning these analyses with Pc37 removed strongly suggested that this individual is not a hybrid (minimum posterior probability of being pure *P. cinereus* among the four analyses conducted with Pc37 removed = 0.98) without yielding any other qualitative changes in the results (not shown).

**FIGURE 8:**
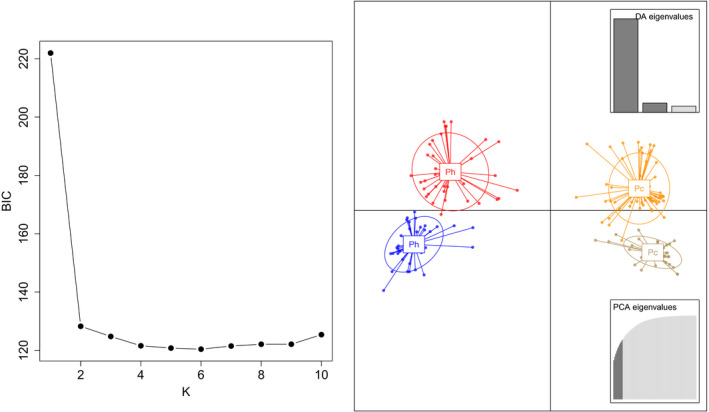
Results of the discriminant analysis of principal components (DAPC), including the quality of K‐means clustering solutions for *K* = 1–10, as assessed by the Bayesian Information Criterion (BIC; left), and ordination of samples within the canonical axes associated with the first two discriminant functions (*df*1 = horizontal axis, *df*2 = vertical axis). Pc = *P. cinereus* and Ph = *P. hubrichti*

Assessment of K‐means clustering solutions for *K* = 1–10 via BIC revealed that BIC decreased until *K* = 4 and leveled off between *K* = 5 and *K* = 9 before modestly increasing at *K* = 10 (Figure 8). As such, we selected *K* = 4 as the best number of clusters to use when summarizing our data with DAPC. The cross‐validation procedure of Jombart and Collins (2015) indicated retention of 10 principal components as optimal, and all three discriminant functions were retained. As can be seen in Figure 8, the first discriminant function primarily separates the species, while the second discriminant function provides additional separation between intraspecific clusters. Moreover, the DAPC unambiguously correctly assigned 100% of genotypes to their morphologically determined species identities. The DAPC results are also consistent with the STRUCTURE results in the sense that membership patterns within the *P. hubrichti* clusters are indicative of modest population structure (cluster 1 = 36 individuals from the contact zone and 2 individuals from Floyd's Field, cluster 4 = 15 individuals from the contact zone and 22 from Floyd's Field). However, assignment patterns within *P. cinereus* clusters are not suggestive of differentiation between the reference site (Thunder Ridge) and sites within the contact zone (cluster 2 = 26 individuals from the contact zone and 9 individuals from Thunder Ridge, cluster 3 = 47 individuals from the contact zone and 13 from Thunder Ridge).

As presented in Table 3, the AMOVA results are generally consistent with the STRUCTURE results, as they reveal pronounced differentiation between species. This finding is reinforced by the pairwise standardized *F*_ST_ values estimated by AMOVA, which show that differentiation between intraspecific populations (*F*′_ST_ between Floyd's Field and the *P. hubrichti* contact zone population = 0.150 and *F*′_ST_ between Thunder Ridge and the *P. cinereus* contact zone population = 0.046) is far lower than for interspecific populations (*F*′_ST_ between Floyd's Field and Thunder Ridge = 0.922, *F*′_ST_ between the two contact zone populations = 0.953, *F*′_ST_ between Floyd's Field and the *P. cinereus* contact zone population = 0.926, and *F*′_ST_ between Thunder Ridge and the *P. hubrichti* contact zone population = 0.956). Lastly, as suggested by STRUCTURE the *F*′_ST_ values obtained by AMOVA make clear that there is more pronounced subdivision within *P. hubrichti* than *P. cinereus*.

**TABLE 3:** Hierarchical variance partition and *F*‐statistics generated by AMOVA

Source	*df*	SS	MS	Est. Var.	% Var.	*F*‐statistic	*p*‐value
Among species	1	274.555	274.555	1.566	41.682	*F*_RT_ = 0.417	.0001
Among populations	2	17.540	8.770	0.093	2.485	*F*_SR_ = 0.043	.0002
Among individuals	166	425.893	2.566	0.468	12.460	*F*_ST_ = 0.442	.0001
Within individuals	170	277.000	1.629	1.629	43.372	*F*_IS_ = 0.223	.0001
Total	339	994.988	N/A	3.757	100.000	*F*_IT_ = 0.556	.0001

Hierarchical partitioning of diversity via the “different is different” approach described by Smouse et al. (2017) is presented in Table 4. Unsurprisingly, the among‐species diversity partition, ignoring subdivision, is over 90% of the maximum level possible, reinforcing the idea that sympatric *P. hubrichti* and *P. cinereus* are strongly differentiated from one another. However, perhaps the most striking feature of this analysis is that scaled diversity metrics are statistically larger in *P. hubrichti* relative to *P. cinereus* at all levels of the hierarchy encapsulated by the partition (Table 4).

**TABLE 4:** QDiver “different is different” diversity partition results

*Plethodon cinereus* Diversity components & statistical tests	Study‐wide average Diversity & statistical tests	*Plethodon hubrichti* Diversity components & statistical tests
	*γ* ^~^ = 0.734	
	*δ* ^~^_AS_ = 0.905 (*p* = .0001)	
*σ* ^~^_WS_ = 0.419	*σ* ^~^_WS_ = 0.518 (*p* = .0001)	*σ* ^~^_WS_ = 0.677
*β* ^~^_AP_ = 0.036	*β* ^~^_AP_ = 0.066 (*p* = .0009)	*β* ^~^_AP_ = 0.142
*α* ^~^_WP_ = 0.413 (*p* = .5700) Thunder Ridge = 0.424 Contact Zone = 0.412	*α* ^~^_WP_ = 0.507 (*p* = .0001) *P. cinereus* = 0.413 *P. hubrichti* = 0.659	*α* ^~^_WP_ = 0.659 (*p* = .3747) Floyd's Field = 0.670 Contact Zone = 0.656
*ɛ* ^~^_AI_ = 0.301	*ɛ* ^~^_AI_ = 0.379 (*p* = .0001)	*ɛ* ^~^_AI_ = 0.544
*ω* ^~^_WI_ = 0.322	*ω* ^~^_WI_ = 0.416	*ω* ^~^_WI_ = 0.511

Note

Scaled diversity cascades are presented along with among strata *p*‐values based on permutation tests and within strata *p*‐values based on Bartlett's test of homogeneity.

Abbreviations: *ɛ* ^~^_AI_, among individuals scaled diversity; *α* ^~^_WP_, within population scaled diversity; *β* ^~^_AP_, among populations scaled diversity; *γ* ^~^, total scaled diversity across the entire study; *δ* ^~^_AS_, among species scaled diversity; *σ* ^~^_WS_, within species scaled diversity; *ω* ^~^_WI_, within individuals scaled diversity.

Locus‐specific values of *D*_EST_ quantifying allelic differentiation between *P. hubrichti* and *P. cinereus* ranged from 0.621 to 1.000 and were highly statistically significant (maximum *p* = .0001), while the overall estimate of *D*_EST_ obtained by summarizing across loci was 0.906 (*p* = .0001). Locus‐specific *G*′_ST_ estimates comparing the *P. hubrichti* reference and contact zone populations ranged from 0.000 to 0.519 and were significant at the 0.05 level for four (Pc7, Pc15, Pc17, Pc22) of eight loci. Similarly, locus‐specific values for *D*_EST_ comparing the *P. hubrichti* reference and contact zone populations ranged from 0.000 to 0.485, with the same four loci exhibiting statistical significance at the 0.05 level. The overall estimate of *G*′_ST_ for *P. hubrichti* obtained by summarizing across loci was 0.139 (*p* = .0001), while the analogous estimate of *D*_EST_ was 0.108 (*p* = .0001). Locus‐specific estimates of *G*′_ST_ comparing the *P. cinereus* reference and contact zone populations ranged from 0.000 to 0.299 and were statically significant at the 0.05 level for four of eight loci (Pc4, Pc17, Pc22, and Pc28). The global estimate of *G*′_ST_ for *P. cinereus* obtained by summarizing across loci was 0.037 (*p* = .0001). Analogous locus‐specific estimates of *D*_EST_ ranged from 0.000 to 0.281 and were statistically significant at the 0.05 level for Pc4, Pc7, Pc22, and Pc28. The overall estimate of *D*_EST_ obtained by summarizing across loci was 0.020 (*p* = .0001).

#### 3.2.3 Gene flow

The analyses that we performed in MIGRATE resulted in a median estimate of *θ* = 7.533 (2.5% = 2.400, 97.5% = 12.267) for the *P. hubrichti* contact zone population and a median estimate of *θ* = 2.467 (2.5% = 0.000, 97.5% = 5.200) for the Floyd's Field population. Assuming a standard microsatellite mutation rate of 5 × 10^–4^ (Garza & Williamson, 2001) these results suggest *N*_e_ = 3,767 for the *P. hubrichti* contact zone population and *N*_e_ = 1,234 for the Floyd's Field population. By comparison, MIGRATE provided a median estimate of *θ* = 2.067 (2.5% = 0.000, 97.5% = 4.400) for the *P. cinereus* contact zone population and a median estimate of *θ* = 2.200 (2.5% = 0.000, 97.5% = 4.800) for the Thunder Ridge population. Thus, assuming µ = 5 × 10^–4^ suggests that *N*_e_ = 1,034 for the *P. cinereus* contact zone population and *N*_e_ = 1,100 for the Thunder Ridge population.

With respect to gene flow, MIGRATE provided a median estimate of 4*N*_e_ *m* = 31.000 (2.5% = 0.000, 97.5% = 66.000) for the *P. hubrichti* contact zone population and a median estimate of 4*N*_e_ *m* = 35.000 (2.5% = 0.000, 97.5% = 74.000) for the Floyd's Field population. Thus, there are just under eight effective migrants arriving in the contact zone from Floyd's Field per generation and just under nine effective migrants arriving in Floyd's Field from the contact zone per generation. By comparison, the median estimate of 4*N*_e_ *m* = 33.000 (2.5% = 0.000, 97.5% = 72.000) for the *P. cinereus* contact zone population and the median estimate is 85.000 (2.5% = 20.000, 97.5% = 144.000) for the Thunder Ridge population. Thus, there are just over eight effective migrants arriving in the contact zone from Thunder ridge per generation and just over 21 effective migrants arriving at Thunder Ridge from the contact zone per generation.

#### 3.2.4 Bottleneck testing

Examination of departures between H_E_ and H_EQ_ based on the seven loci that were variable in Floyd's Field did not reveal any statistical evidence of heterozygote excess or deficiency in this population. Similarly, the analyses we performed in BOTTLENECK did not reveal any evidence of heterozygote excess or deficiency in the *P. hubrichti* or *P. cinereus* contact zone populations. However, there was marginal evidence of heterozygote deficiency in the *P. cinereus* reference population (THRID; one‐tailed Wilcoxon test = 0.037).

#### 3.2.5 Demographic modeling

We estimated a demographic history for the contact zone and reference populations using approximate Bayesian computation implemented in DIYABC, under a simple model (Figure 2). The median posterior estimates of *N*_e_ were 3,451 and 7,915 for the *P. hubrichti* reference and contact zone populations, respectively. The median posterior estimates of *N*_e_ for *P. cinereus* were 4,811 for the reference site and 2,057 for the contact zone. The divergence time estimated for the *P. cinereus* reference and contact zone populations (90 generations, 90% highest density probability interval (HDPI) = 1–369 generations) was shorter than the estimated divergence of the *P. hubrichti* populations (585 generations, 90% HDPI = 212–992 generations), which, along with analyses of genetic diversity and divergence, is consistent with a more recent expansion of *P. cinereus* into the contact zone, though the 90% HDPIs do overlap.

### 3.3 Homing experiment

**FIGURE 9:**
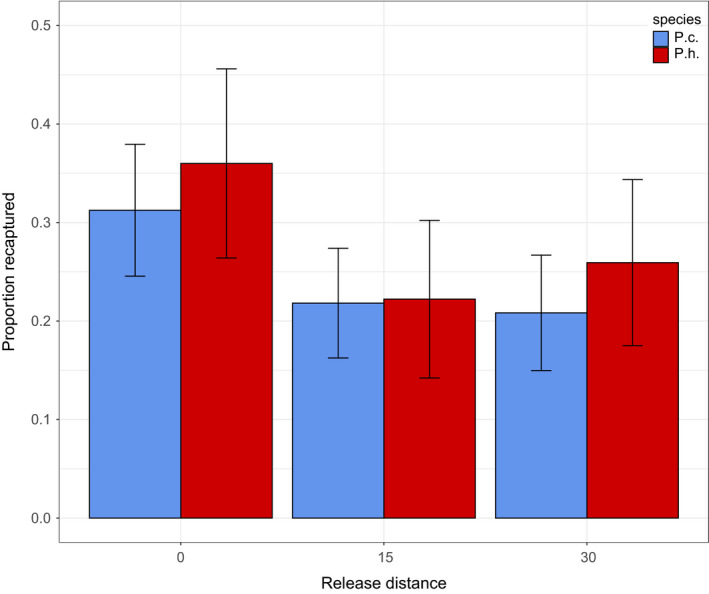
Return and recapture rates from the homing experiment. *Plethodon* *cinereus* (P.c.) and *P. hubrichti* (P.h.) were either returned to their original site of capture (Release distance = 0 m) or displaced 15 m or 30 m in a randomly chosen direction. The proportion of salamanders recaptured underneath their home cover object is shown along with standard errors estimated from a binomial distribution

We marked and released a total of 79 *P. hubrichti* and 151 *P. cinereus*. For controls that were marked and replaced at their site of capture, recapture rates at the original capture location were 36% (9 of 25) and 31% (15 of 48), respectively. Overall, recapture rates for displaced salamanders were about two‐thirds of controls, suggesting that about this proportion returned to their original cover objects (Figure 9). In three cases, salamanders appear to have lost one elastomer tag, but in each of these cases the salamander was identifiable based on the remaining three tags.

Recapture rates were very similar for the two species at both release distances. For salamanders displaced 15 m (Figure 9), the recapture rate was 22% for both *P. hubrichti* (6 of 27) and for *P. cinereus* (12 of 55). For salamanders displaced 30 m (Figure 9), the recapture rate was 26% for *P. hubrichti* (7 of 27) and 21% for *P. cinereus* (10 of 48). Based on logistic regression, there was no significant difference in return frequency between species (*b* = 0.15, *SE* = 0.40, *p* = .70) and no significant difference in return frequency between release distances (*b* = 0.036, *SE* = 0.38, *p* = .92).

Salamanders were only rarely found under cover objects other than those from which they were originally captured. One *P. hubrichti* control was captured 12 m away from its original cover object, and one *P. hubrichti* released at 15 m was recaptured 8 m away from its original cover object. Similarly, one *P. cinereus* released at 15 m was captured under a board 12.6 m away from its original cover object, close to its release location. Thus, it does not appear that our analysis of return rate was substantially influenced by captures of salamanders from under cover objects other than the original capture location.

## 4 DISCUSSION

Range shifts due to climate change can lead to increased hybridization and the potential for genetic swamping of range‐restricted species (Muhlfeld et al., 2014; Walls, 2009). Although hybridization regularly occurs within clades of the woodland salamander genus *Plethodon* (Highton, 1995; Lehtinen et al., 2016; Weisrock, Kozak, & Larson, 2005), in this study we found little, if any, evidence for hybridization between a mountaintop endemic species (*P. hubrichti*) and its widespread congener (*P. cinereus*). Based on multilocus genotypes for eight microsatellites, all individual salamanders could be assigned to species with a probability > .8, and in each case these assignments matched our initial classification based on morphological features (e.g., coloration of the venter and coloration of the dorsal stripe). Our analysis was based on 124 salamanders from within the contact zone, including 18 individuals that were specifically sampled for having unusual color patterns. From these sample sizes, we cannot rule out hybridization at a low frequency, though any hybridization would appear to be, at most, rare. Thus, from a conservation perspective, our results do not find that hybridization presents an urgent threat to *P. hubrichti*.

Whereas *P. cinereus* do not appear to be hybridizing regularly with *P. hubrichti*, several lines of evidence suggest that they may be expanding into the range of *P. hubrichti*. We found that *P. hubrichti* in and around the contact zone had higher levels of genetic diversity than did *P. cinereus* at all levels within our hierarchical diversity partition. In addition, every method we employed indicated that differentiation between populations across the contact zone was greater for *P. hubrichti* than for *P. cinereus*. Elsewhere in Virginia, *P. cinereus* displays much higher levels of genetic diversity in microsatellite alleles and higher differentiation among populations over similar spatial scales (Cabe et al., 2007; Cameron et al., 2017). The comparatively low levels of diversity and differentiation for *P. cinereus* at our study sites are consistent with the hypothesis that these populations represent a relatively recent range extension of *P. cinereus* into areas formerly occupied by *P. hubrichti*. This interpretation is also consistent with the recent phylogeographic analysis of *P. cinereus* by Radomski, Hantak, Brown, and Kuchta (2020), which showed that our study area is near the border between two distinct *P. cinereus* clades. Thus, continued population expansion by *P. cinereus* could represent a critical threat to the long‐term persistence of *P. hubrichti*, particularly given its small range and its proximity to high‐density populations of *P. cinereus*. We suggest that continued monitoring in conjunction with the *P. hubrichti* conservation plan should include data collection on the range limits of *P. cinereus* in the Peaks of Otter region so that continued range expansion can be detected.

The similarity of movement ability between the two species as documented by the homing experiment also potentially bears on the question of range limitation in *P. hubrichti*. Bernardo et al. (2007) suggested that mountaintop endemic salamanders may evolve lower metabolic rates as an adaptation to high‐elevation environments, leading to reduced dispersal ability and increased genetic differentiation among populations. Although we did find higher levels of genetic differentiation in *P. hubrichti* as compared to *P. cinereus*, results of the homing experiment, as well as the analyses we performed in MIGRATE, suggest that this difference does not result from marked differences in dispersal ability between *P. hubrichti* and *P. cinereus*. In addition to having movement rates similar to those of *P. cinereus*, recent physiological research suggests that *P. hubrichti* has metabolic rates that are similar to those of *P. cinereus*, particularly at higher temperatures (Markle & Kozak, 2018). Thus, we believe that population history (i.e., longer residency of *P. hubrichti* vs. *P. cinereus* in the region) is a better‐supported explanation for higher levels of differentiation in *P. hubrichti* at our study sites than is reduced dispersal ability.

Although our study does not formally address the nature of competition between *P. cinereus* and *P. hubrichti*, competition and competitive displacement appear to be common phenomena among similarly‐sized *Plethodon* (Griffis & Jaeger, 1998; Hairston, 1951; Highton, 1995). Arif, Adams, and Wicknick (2007) suggested, based on an ecological niche model, that *P. cinereus* should be able to occupy most of the range of *P. hubrichti* and that only competition from *P. hubrichti* has prevented their spread. Furthermore, Brophy and Reichenbach (2020) showed that removal of *P. cinereus* increased surface activity of *P. hubrichti,* and Marsh et al. (2020) found that fitness correlates of both *P. cinereus* and *P. hubrichti* were reduced in the zone of contact between them. The hypothesis that competition has slowed or limited the spread of *P. cinereus* is not inconsistent with the hypothesis that the *P. cinereus* range has spread in the past and may continue to spread in the future. It is possible that *P. cinereus* tend to outcompete *P. hubrichti* at lower elevations, and that *P. cinereus* continue to displace *P. hubrichti* along the range margin, albeit at a rate too slow to detect in ecological studies (Aasen & Reichenbach, 2004). The strength of competition between the species may also be reduced by differences in microhabitat preferences between them. For example, Farallo and Miles (2016) found evidence from multidimensional scaling for complex microhabitat differences between *P. hubrichti* and *P. cinereus*, and Reichenbach and Kniowski (2009) observed that juvenile (but not adult) *P. hubrichti* were more commonly found under rocks compared to *P. cinereus*. Recent studies of other endemic *Plethodon* in the region have similarly highlighted the role of microhabitat in explaining species distributions (Amburgey, Miller, Brand, Dietrich, & Grant, 2020).

Our study has several important limitations. Most notably, while eight microsatellite markers and one mitochondrial gene allowed us to document basic patterns (e.g., little to no evidence for hybridization, likely expansion of *P. cinereus*), these markers had limited resolution for evaluating more detailed demographic scenarios. In principle, population genomic approaches (e.g., Weisrock et al., 2018) could allow us to better estimate the rate of spread of *P. cinereus* and the rate of population growth within the contact zone. Second, our study was restricted to the high‐elevation contact zone at the northeastern edge of the range of *P. hubrichti*. We selected this area because both species reach high densities here, facilitating greater ease of collecting and potentially increasing the likelihood of hybridization. At the lower elevation limits of *P. hubrichti*, both species tend to be uncommon, so sufficient samples sizes would be difficult to obtain. Interactions between the two species, including whether they hybridize and whether *P. cinereus* is spreading, could be different at these lower elevation sites. Indeed, other related species *of Plethodon* are known to hybridize at some locations but not at others (Carpenter, Jung, & Sites, 2001; Highton, 1995). From a conservation perspective, one could argue that the high‐elevation contact zone is of particular concern since it contains much higher densities of *P. hubrichti* and therefore spread of *P. cinereus* here might be more detrimental to the long‐term persistence of the mountaintop endemic. Alternatively, low elevation sites might be particularly important for the range expansion of *P. cinereus*, particularly in the presence of climate change. A third limitation of our study is that using only a single reference site could limit our inferences about hybridization. Assignment probabilities based on microsatellite alleles would be altered if other alleles were present at unsampled sites near the contact zone. As a result, we cannot rule out hybridization based on the generally high assignment probabilities of salamanders in our specific samples. Ultimately, it would require more extensive sampling of *P. cinereus* and *P. hubrichti* in the region to rule out occasional hybridization at this site or at other locations.

The results of the homing experiment should also be interpreted with caution. *Plethodon cinereus* have previously been shown to home across distances as far as 90 m (Kleeberger & Werner, 1982), though landscape barriers such as roads and streams tend to reduce return rates (Marsh et al., 2005, 2007). Although we interpret similar homing ability in *P. cinereus* and *P. hubrichti* as indicative of similar overall dispersal ability, this interpretation is not necessarily correct. For example, salamander species may have the ability to home when displaced, but nevertheless disperse from their natal site at different frequencies that depend on behavioral factors such as territoriality or mate‐seeking behavior, and it is dispersal under natural conditions that will determine patterns of genetic differentiation. Additionally, homing rates for both species in the experiment did not decline between the 15‐m and the 30‐m treatments, suggesting that the greater distance did not present a challenge for either species. It therefore remains a possibility that over greater distances, differences in return rates between the two species would have been observed.

In spite of these limitations, our results have yielded some novel insights about the population genetics and evolutionary history of the mountaintop endemic salamander, *P. hubrichti*. In particular, we find that genetic diversity is quite high in *P. hubrichti*, which is perhaps surprising for a species with one of the smallest ranges of all vertebrates in mainland North America. We believe that two factors may have contributed to the high genetic diversity of *P. hubrichti*. First, despite their small range, population densities of *P. hubrichti* are locally high, leading to high effective population sizes. We estimated *N*_e_ as between 3,700 (MIGRATE) and 7,915 (DIYABC) for the contact zone population and 1,234 (MIGRATE) and 3,451 (DIYABC) for the reference population. These populations are not isolated, but rather continuous with other *P. hubrichti* populations, so these values would typically be large enough for populations to avoid losing genetic variation via drift (Reed, 2005; Shaffer, 1981). The second factor, which may have contributed to *P. hubrichti's* genetic diversity, is the evolutionary history of the species. The closest relative of *P. hubrichti* is the Cheat Mountain Salamander, *P. nettingi*, another high‐elevation endemic which is found in remnant spruce forests in West Virginia about 150 km away on the other side of the Shenandoah Valley (Kozak, Weisrock, & Larson, 2005; Sites, Morando, Highton, Huber, & Jung, 2004). The two species likely diverged during drier periods of the Pliocene when their distributions were restricted to moister forest habitats at higher elevations (Highton, 1995; Kozak & Wiens, 2006). Assuming this scenario is correct, the common ancestor of *P. hubrichti* and *P. nettingi* would have been widespread, facilitating the generation and maintenance of high genetic diversity. Although the long‐term population consequences of genetic diversity are not fully understood (Hadly, van Tuinen, Chan, & Heiman, 2003; Reed, 2010), in general, genetic diversity appears to contribute to long‐term population persistence (Frankham, 2005; Pearman & Garner, 2005), and similarly, low genetic diversity can hasten extinction (O'Grady et al., 2006; Saccheri et al., 1998). All else being equal, the high genetic diversity of *P. hubrichti* would be expected to prove useful as the species responds to continued climate change and the potential expansion of *P. cinereus* into its range.

## CONFLICT OF INTERESTS

The authors declare that they have no conflicts of interests or competing interests.

## AUTHOR CONTRIBUTION

**Robert B. Page:** Conceptualization (supporting); Data curation (equal); Formal analysis (lead); Funding acquisition (equal); Methodology (lead); Project administration (equal); Resources (equal); Software (lead); Supervision (equal); Validation (lead); Visualization (lead); Writing‐original draft (equal); Writing‐review & editing (equal). **Claire Conarroe:** Data curation (supporting); Investigation (equal). **Diana Quintanilla:** Data curation (supporting); Investigation (equal). **Andriea Palomo:** Data curation (supporting); Investigation (equal). **Joshua Solis:** Data curation (supporting); Investigation (equal). **Ashley Aguilar:** Data curation (supporting); Software (supporting); Visualization (supporting). **Kelly Bezold:** Investigation (supporting); Project administration (supporting); Supervision (supporting). **Andrew M. Sackman:** Formal analysis (supporting); Software (supporting); Visualization (supporting). **David M. Marsh:** Conceptualization (lead); Data curation (equal); Formal analysis (supporting); Funding acquisition (equal); Methodology (supporting); Project administration (equal); Resources (equal); Software (supporting); Supervision (equal); Validation (supporting); Visualization (supporting); Writing‐original draft (equal); Writing‐review & editing (equal).

## Data Availability

The microsatellite and homing datasets are deposited in Dryad (https://doi.org/10.5061/dryad.8gtht76mg). DNA sequences are deposited in GenBank.
